# *trans*-Resveratrol as A Neuroprotectant 

**DOI:** 10.3390/molecules15031196

**Published:** 2010-03-03

**Authors:** Ellen L. Robb, Jeffrey A. Stuart

**Affiliations:** Department of Biological Sciences, Brock University, St. Catharines, ON, L2S 3A1, Canada; E-Mail: ellen.robb@brocku.ca

**Keywords:** resveratrol, mitochondria, MnSOD, superoxide dismutase, reactive oxygen species, ROS, estrogen, phytoestrogen, stress resistance, neuroprotection

## Abstract

Epidemiological evidence indicates that nutritionally-derived polyphenols such as resveratrol (RES) have neuroprotective properties. Administration of RES to culture media protects a wide variety of neuronal cell types from stress-induced death. Dietary supplementation of RES can ameliorate neuronal damage and death resulting from both acute and chronic stresses in rodents. The specific molecular mechanisms by which RES acts at the cellular level remain incompletely understood. However, many experimental data indicate that RES reduces or prevents the occurrence of oxidative damage. Here we discuss possible mechanisms by which RES might exert protection against oxidative damage and cell death. Evidence suggesting that RES’s chemical antioxidant potential is not sufficient explanation for its effects is discussed. Putative biological activities, including interactions with estrogen receptors and sirtuins are critically discussed. We provide a synthesis of how RES’s phytoestrogenic properties might mediate the neuronal stress resistance underlying its observed neuroprotective properties.

## 1. Introduction

The efficacy of nutritionally derived compounds as neuroprotective agents is increasingly supported by empirical evidence. Plant-derived molecules including polyphenols have demonstrated neuroprotective activities in cell culture and animal models. However, the molecular mechanisms that give rise to their protective effects are generally not well understood. Elevated levels of oxidative damage and an increased occurrence of cell death are common observations in chronic neurodegenerative diseases and acute ischemic injury. Polyphenols are small molecule antioxidants that are hypothesized to offer protection against the negative effects of oxidative stress in many tissues including brain, and this may underlie their ability to protect against cell death. Although dietary sources of antioxidants are extensive, a great deal of research interest has been directed towards the compounds found in red wine.

Resveratrol (RES), a compound found in high concentrations in red wine, and related polyphenols, have inherent antioxidant capacity due to their chemical structure. Direct antioxidant properties were once posited to be responsible for their broad range of biological effects; however, limited bioavailability and relatively weak scavenging abilities make this unlikely. An alternative to direct chemical interactions is the possibility that RES and related polyphenols work to enhance endogenous intracellular defense systems and in turn protect against cellular stress, dysfunction and death. 

We begin this review by first summarizing the literature supporting the neuroprotective actions of RES, followed by a critical review of recent findings regarding putative mechanisms of RES’s actions at the cellular level. We then extend this discussion to dietary delivery strategies that may increase RES’s bioavailability and thereby maximize its neuroprotective effects.

## 2. RES as a Neuroprotectant—Experimental Evidence

RES’s neuroprotective activity has been demonstrated in a wide range of different experimental models including neuronal cell cultures and live animals (Table 1). Experiments with neuronal cell lines are too numerous to summarize here, so only selected examples are described. Micromolar concentrations of RES have been shown to prevent apoptotic cell death in cultured cerebellar [1] and dopaminergic [2] neurons exposed to MPP^+^, a model of Parkinson-like neurodegeneration. Similarly, RES protects rat brain hippocampal slices against oxygen and glucose deprivation, a model of ischemic brain injury. *In vivo*, dietary administration of RES to mice [6] or rats [12] confers protection against acute brain injury caused by transient middle cerebral artery occlusion or cardiac arrest, respectively. Delivery of RES in the diet also protects mice [7] and rats [9] against 1-methyl-4-phenyl-1,2,3,3-tetrahydropyridine (MPTP)- and 6-hydroxydopamine (6-OHDA)-induced neurodegeneration in experimental models of Parkinson’s disease. Dietary RES supplementation reduces plaque formation in brains of a transgenic mouse model of Alzheimer’s disease [11]. In the primate species *Microcebus murinus* chronic dietary administration of RES, for up to 18 months confers improved performance in a variety of behavioural tests [13]. Thus, regular dietary supplementation with RES can provide neural protection against a variety of potentially cytotoxic stresses and ameliorates neurodegeneration resulting from both acute and chronic insults.

**Table 1:** Evidence for resveratrol as a neuroprotectant.

Model	Treatment	Stressor	Effect	Reference
Cerebellar granule neurons	24 h of 1-100 µM RES	MPP+	Antiapoptotic	[1]
PC12 cells	3 h of 100 µM RES	MPP^+^	Antiapoptotic	[2]
SH-SY5Y cells	1 h of 5 µM RES	Dopamine	Antiapoptotic	[3]
SH-SY5Y cells	24–72 h of 1–50 uM RES	H_2_O_2_, paraquat or menadione	Reduced incidence of cell death	Robb and Stuart unpublished
PC12 cells co-cultured with N9 microglia	27h of 100 µM RES	LPS treatment	Antiapoptotic	[4]
Rat hippocampal slices	Acute 100 µM RES	Oxygen-glucose deprivation	Neuroprotective	[5]
Mice	50 mg/kg/day gavage	Middle cerebral artery occlusion	Neuroprotective	[6]
Mice	50–100 mg/kg/day	MPTP	Prevented loss of DA neurons	[7]
Mice	Acute RES at 30 mg/kg i.v.	MPTP	Reduced oxidative damage; protected DA neurons	[8]
Mice	10–40 mg/kg/d for 10 wks	6-OHDA	Reduced neuronal damage	[9]
Inducible p25 Alzheimer’s mouse model	2.5 µg RES injected into lateral brain ventricles	N.A.	Neuroprotective	[10]
Tg 19959 mouse Alzheimer’s model	300 mg/kg/d RES in diet	N.A.	Reduced plaque pathology	[11]
Rats	10–100 µM RES by i.p. injection	Asphyxial cardiac arrest	Neuroprotective	[12]

### 2.1. RES ameliorates oxidative stress

Many different conditions of cellular stress are united by a common observation of oxidative damage. In both acute brain injuries [14] and chronic neurodegenerative diseases [15], increased levels of cellular oxidative damage are typically evident. Oxidative stress is broadly defined as a perturbation of cellular homeostasis such that the rate of reactive oxygen species (ROS) production exceeds that of their neutralization. If homeostasis is not re-established, oxidative stress may progress toward the initiation of apoptotic cell death and tissue degeneration. There is abundant evidence that RES plays a role in attenuating this process via its ability to ameliorate oxidative stress.

In neurons, ROS are produced primarily from the mitochondrial electron transport chain as electrons pass from reduced complexes directly to molecular oxygen. The resulting superoxide anion participates in a variety of reactions that produce hydrogen peroxide, hydroxyl radical and peroxynitrite, which are subsequently involved in the oxidation of DNA, proteins and membranes. An aberrant overproduction of mitochondrial ROS is thought to contribute to both acute and chronic neuronal stress and death. For example Parkinson’s disease, which is characterized by the specific loss of dopaminergic neurons, can be induced by injection of 1-methyl-4-phenyl-1,2,3,6-tetrahydropyridine (MPTP) [7,8]. This toxin is converted to MPP+ in satellite cells, and then taken up by dopaminergic neurons and electrophoretically accumulated within the mitochondrial matrix where it inhibits respiratory complex I [16] and increases superoxide production. Isolated mitochondria from rats treated with rotenone, a similar model of Parkinsonism, display increased levels of superoxide production, as well as an increased propensity to undergo membrane permeability transition pore opening [17]. In these Parkinson’s disease models, treatment with mitochondria-targeted antioxidants, such as Szeto-Schiller peptide and MitoQ, can prevent acute neurological injury and neurodegeneration [reviewed in 18]. Szeto-Schiller peptide SS-31 is a short peptide containing aromatic and basic amino acids that accumulates in mitochondria and may reduce ROS concentrations [19]. SS-31 protects neuronal cell lines against exogenous oxidants [20]. It has recently been shown to protect against MPTP neurotoxicity in mice in a dose dependent manner [21]. Thus, rodent models of acute neuronal injury and chronic neurodegenerative disease indicate an important role for mitochondria-derived ROS, and provide evidence for the benefits of mitochondria-targeted antioxidants.

Abundant evidence from neuronal cell culture experiments and *in vivo* models indicates that RES-mediated neuroprotection occurs concomitantly with measureable reductions in the oxidative damage load. For example, RES reduces oxidative DNA damage in C6 Glioma cells exposed to hydrogen peroxide [22]. RES reduces stress-induced ROS production in cerebellar neurons [1]. *In vivo,* RES has been shown to decrease various biomarkers of cellular oxidative damage, including DNA oxidation and lipid peroxidation [23,24,25,26,27,28]. Taken together, these results support the interpretation that RES confers neuroprotection due to its activity as an antioxidant. However, it appears that the ability to induce the expression of endogenous antioxidant enzymes, rather than its chemical antioxidant activity *per se,* underlies the antioxidant capacity of RES.

### 2.2. RES as a chemical antioxidant

The neuroprotective actions of RES and related phenolics at the cellular and organism level have been attributed to their chemical antioxidant capacities. *In vitro,* RES may efficiently neutralize ROS via the donation of a hydrogen atom and indeed, RES does protect cultured cells against death induced by a variety of exogenous oxidative stressors [29,30,31]. The extrapolation of *in vitro* results to *in vivo* systems is cautioned by a number of important experimental limitations. *In vitro* studies of antioxidants often employ supraphysiological concentrations, and may be complicated by the potential of some compounds to act as pro-oxidants *in vitro*, generating ROS as they decompose or react with surrounding molecules (see [32] for a review). These pro-oxidants cause mild oxidative stress which induces cellular defense systems and in turn increases the ability to withstand subsequent stressors (i.e., hormesis). This is particularly important in the context of ischemia-reperfusion injury as exposure to a low grade, non-lethal stressor invokes the expression of enzymes capable of protecting against more severe stress. Currently, however, there is no evidence to support the hypothesis that RES generates significant quantities of ROS in cell culture media. Ungvari *et al*. found that human coronary arterial endothelial cells cultured in the presence of micromolar concentrations of RES produced lower levels of superoxide and hydrogen peroxide than control treated cells [33]. While the reduction in ROS production observed likely arises due to an increase in ROS detoxification, the data do not support a prooxidant role for RES.

*In vitro*, the location of an antioxidant compound is also an important consideration, as its effects will be different if it is sequestered in the culture media or accumulates within cells. MitoQ, a hydrophobic antioxidant synthesized with a mitochondrial targeting sequence [34], partitions in mitochondrial membranes and has been shown to protect against oxidative damage following ischemia-reperfusion injury in rats [35]. Similarly, RES is hydrophobic and may accumulate in membranes at concentrations sufficient to act as an antioxidant following chronic exposure. There is evidence for an association between RES and mitochondria, including direct interactions with electron transport chain complexes [36]. Interestingly, RES’s protective effect against a range of exogenous stressors, including oxidants, *in vitro* persists when RES is withdrawn from the culture media prior to the addition of exogenous oxidants [37], suggesting that RES is not acting to buffer ROS in the culture media. It is unknown whether RES accumulates within cells to act as an antioxidant or if its biological activities are responsible for its ability to enhance cellular stress resistance. Further research into the intracellular localization and accumulation of RES is required to understand its mechanism of action within cells. 

In contrast to many chemical antioxidants, RES has been reported to interact with a wide range of cellular systems with the potential to protect against cell death, and it is therefore unclear whether direct chemical antioxidant or biological activities are responsible for RES’s ability to protect against oxidative stress*in vitro* [38]. In contrast, it is quite clear that i*n vivo* RES’s chemical antioxidant properties are unlikely to be important. Concentrations of RES necessary to achieve protective effects *in vitro* are supraphysiological, and extrapolation of RES’s *in vivo* effects from these experiments is not possible. It is interesting to note that RES is a fairly weak antioxidant, and radical scavenging would likely be observed only at concentrations above 0.1M [39,40]. Plasma concentrations of RES in rodents following a pharmaceutical dose are typically in the low micromolar range, far below the concentrations required for chemical antioxidant effects [41]. In humans, an oral dose of 25mg only yields plasma concentrations of free RES in the low nanomolar range [42]. Additionally, RES and related phenolics are quickly metabolized to glucuronidated forms with reduced antioxidant capacities [43]. Given the low bioavailability of RES and the rapid appearance of its metabolites, it seems unlikely that RES’s chemical antioxidant properties are important to its beneficial effects *in vivo*. 

In spite of the low probability that RES can act as a chemical antioxidant *in vivo*, it does appear to ameliorate conditions associated with oxidative stress. Below we explore the hypothesis that RES protects against oxidative stress via an upregulation of antioxidant enzymes.

## 3. RES and MnSOD

Mitochondria possess endogenous defense systems to protect against oxidative stress. These systems include antioxidant enzymes that detoxify radicals and repair enzymes that maintain macromolecule integrity. Within this review, we will primarily focus on the mitochondrial antioxidant enzyme MnSOD, a nuclear encoded protein that is targeted to the mitochondria. Within the matrix MnSOD converts the superoxide anion to hydrogen peroxide which is further detoxified by the enzymes glutathione peroxidase (in mitochondria) or catalase (in cytosol following diffusion of hydrogen peroxide out of mitochondria).

Transgenic overexpression of the mitochondrial superoxide dismutase MnSOD, is protective against oxidative stress in a wide variety of experimental contexts [44,45,46,47]. *In vitro*, MnSOD is capable of protecting neuronal cells from various oxidative stressors. Pheochromocytoma PC6 cells overexpressing MnSOD maintain mitochondrial membrane potential, intracellular ATP levels, and are resistant to apoptosis induced by a range of chemical stressors, including the Parkinson’s mimetic MPTP [44]. Mattson *et al*. hypothesized that MnSOD induction protects hippocampal neurons from oxidative stress induced apoptosis *in vitro* by reducing membrane peroxidation [48]. 

The importance of MnSOD to neuron health is clearly supported by *in vivo* evidence from animal models. In mice, deletion of the MnSOD gene causes severe symptoms from birth including neurological defects, limiting lifespan to several weeks [49,50]. MnSOD^+/-^ mice show increased incidence of apoptotic cell death induced by oxidative stress in tissues including brain [51,52]. In contrast, overexpression of MnSOD in mice provides protection from stress induced cell death, including neurons. Transgenic mice that overexpress human MnSOD are protected from oxidative damage and neuron loss induced by ischemia and the neurotoxin MPTP [44]. In brain, elevation of MnSOD has been shown to attenuate neuron cell death in the event of oxidative stressors such as MPTP and 6-hydroxydopamine [53,54]. In transgenic mice that overexpress MnSOD, cortical neuronal apoptosis is reduced following chronic intermittent hypoxia. This is accompanied by a reduction in spatial learning deficits in the stressed mice [55]. A similar observation was recently reported in a transgenic murine model of Alzheimer’s disease in which MnSOD was overexpressed [56]. It is clear from both *in vivo* and *in vitro* studies that MnSOD has an important role in neuroprotection. Below we explore the hypothesis that MnSOD is an important target of RES, and that many of the effects observed with the overexpression of MnSOD are similarly observed with RES.

### 3.1. MnSOD is a target of resveratrol

MnSOD was initially identified as an important target of RES following the observation that chronic treatment of normal human lung fibroblasts with micromolar concentrations of RES elicits a significant increase in the protein level and activity of MnSOD [57]. A similar increase in MnSOD has been reported in other cell lines treated with RES including coronary artery endothelial cells [33], aortic smooth muscle cells [58], and SH-SY5Y neuroblastoma cells [37]. *In vivo,* RES given in a high fat diet (200 mgRES/kg/day) increases MnSOD in brain tissue of mice [59]. The observation of increased MnSOD *in vivo* is a potential explanation of RES’s neuroprotective effects as many similarities exist with MnSOD overexpression. For example, mice administered RES in their diet were found to be more resistant to MPTP induced dopaminergic neuron death, a finding that is consistent with increased MnSOD capacity in brain [7].

The implication of MnSOD as a specific target of RES both *in vivo* and *in vitro* has not yet been fully realized, but it may represent a critical mechanism behind RES’s beneficial effects on human health. However, RES has been reported to interact with numerous signalling pathways that may be responsible for its broad range of cellular effects including an increase in MnSOD. Understanding the mechanism that leads to increased MnSOD and cellular stress resistance will be a substantial contribution to RES research.

### 3.2. RES as a phytoestrogen – induction of MnSOD

A potential explanation for RES’s cellular effects may be its action as a phytoestrogen, plant derived compounds that mimic the activity of 17-β-estradiol (E2). RES, like the soy product genistein and other polyphenols is structurally similar to E2, with free hydroxyl groups and phenolic ring structures that are important in estrogen receptor binding [60]. RES interacts directly with estrogen receptors alpha and beta with Ki values of 4.33–7.9 micromolar and 7.12–23.73 micromolar respectively, though it appears to exert particularly strong transcriptional effects via ERβ [61; reviewed in 62]. Interestingly, both mitochondria and MnSOD are major downstream targets of E2 signaling ([63]; for A review see [64]). Borras *et al*. showed in MCF7 cells that E2 signaling enhances transcription of MnSOD and glutathione peroxidase (GPx), although the authors did not state whether a mitochondrial GPx isoform (1 or 4) was probed [63]. However, the authors did not correct their results for mitochondrial abundance, an important factor to consider when measuring the level and activity of mitochondrial proteins. Borras *et al*. also reported that mitochondria from females contained higher levels of MnSOD than males [65]. In addition to its reported ability to stimulate mitochondrial antioxidant enzymes, E2 is associated with mitochondrial biogenesis and the transcriptional regulation of components of the electron transport chain (see [66] for A review). Further research into estrogen mediated change in antioxidant capacity with respect to mitochondrial abundance is required. 

Neither MnSOD nor GPx gene promoters contain estrogen response elements, suggesting the E2 induction of their transcription by phytoestrogens is indirect. Borras *et al*. [65] provided evidence for the involvement of the signal transduction pathway mediated by MAP kinase, ERK1/2 and NFκB. UO126, an inhibitor of MAP kinase phosphorylation, eliminated the effects on ROS detoxification observed in MCF-7 cells treated with E2. NFκB is a target of MAP kinases, and is capable of upregulating both MnSOD and GPx. Inhibition of NFκB prevented an increase in MnSOD and GPx in MCF-7 cells treated with E2, supporting its role in the E2 signaling cascade responsible for increases MnSOD [63].

**Figure 1:**
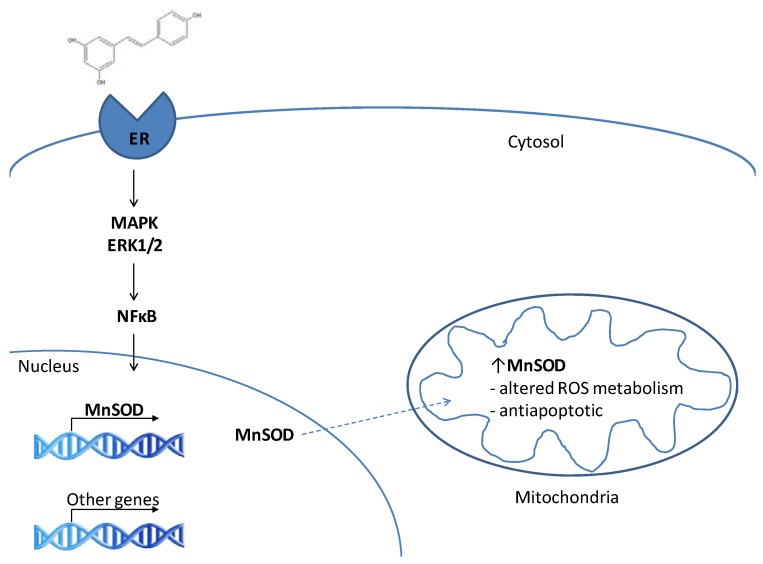
A proposed pathway for the RES-induced stimulation of MnSOD transcription via estrogen receptor-mediated regulation of NFκB activity. Based on the E2-induced transcriptional upregulation of MnSOD and other mitochondrial proteins described by Borras *et al*. [63]. MAPK = mitogen activated protein kinase; ERK1/2 = extracellular signal regulated kinases ½; NFκB = nuclear factor κB; MnSOD = manganese superoxide dismutase.

Interestingly, E2 demonstrates neuroprotective effects similar to those observed with RES. Preincubation of dopaminergic neurons with E2 affords protection against superoxide anions produced by xanthine oxidase, and micromolar concentrations hydrogen peroxide [67]. *In vivo,* E2 has the capacity to prevent the loss of aconitase activity, a superoxide sensitive mitochondrial enzyme, in brain tissue of both male and female rats [68], an observation that is consistent with an increase in MnSOD activity. MnSOD overexpression alone is sufficient to confer neuroprotection in mice [56]. We propose that RES acts both *in vitro* and *in vi*vo by direct agonistic activity with ERβ, and probably to a lesser extent ERα, to stimulate NFκB mediated MnSOD transcription (Figure 1). Robb *et al*. demonstrated a progressive stimulation of MnSOD protein levels and activities in human lung fibroblasts (MRC5) incubated with 50 µM RES [57]. This result has subsequently been repeated in C2C12 murine myoblasts and SH-SY5Y cells [37] and also shown in mouse brain tissue following dietary RES supplementation [59]. In culture, the RES-induced upregulation of MnSOD is associated with increased stress resistance in all three cell lines indicated above [37]. This is consistent with the interpretation that the RES-induced MnSOD upregulation plays a key role in preventing apoptotic death of neurons.

## 4. RES and Sirtuins

RES has gained considerable attention recently for its reported ability to activate sirtuins, a family of protein deacetylases implicated in lifespan regulation of organisms including yeast and worms [38]. Seven sirtuin isoforms have been identified in mammals, each with an apparently unique set of intracellular targets [69]. RES has been reported to activate SIR2 in yeast, and SIRT1 in mammals [70,71,72,73], and this has been offered as an explanation for many of RES’s reported effects.

A neuroprotective activity of sirtuins has been widely suggested. However, while some experimental data support this, there are significant contradictions in the literature (see [74] for a review). Inhibition of SIRT1 activity with sirtinol negates the ability of a RES pre-treatment to protect SK-N-BE neuroblastoma cells from oxidative stress in culture, and hippocampal neurons from ischemic injury in a rat model of stroke secondary to cardiac arrest [12]. SIRT1 overexpression similarly has been shown to confer neuroprotection in a rodent model of Alzheimer’s disease [10]. However, other authors have reported that sirtuins are dispensable for RES-mediated neuroprotection. Sirtinol inhibition of SIRT1 did not attenuate RES-mediated resistance to oxidative stressors in cerebellar granule neurons (CGN) in culture [1], and similarly had no effect on RES’s ability to ameliorate plaque formation in a transgenic model of Alzheimer’s disease [11]. The apparent contradiction between studies might be explained by an incomplete inhibition of SIRT1 at the sirtinol concentration used in some instances. However, Pfister *et al.* used two additional SIRT1 inhibitors, nicotinamide and splitomycin, and still failed to attenuate the RES-mediated protection of cultured CGN from oxidative stress [75]. In contrast, these authors demonstrated that, while SIRT1 overexpression was neuroprotective, this was independent of the enzyme’s deacetylase activity as it was observed even when catalytically dead SIRT1 was expressed [75]. In addition to these negative results, two reports indicate that SIRT1 may actually sensitize neurons to cell death. Li *et al.* demonstrated that both pharmacological SIRT1 inhibition and SIRT1 gene knockout improved neuronal survival following exposure to some oxidative stressors [76]. Liu *et al*. similarly showed that SIRT1 inhibition reduced cell death in response to an excitotoxic insult, noting that intracellular NAD+ levels were maintained when SIRT1 was inhibited [77]. These observations were interpreted as a SIRT1-mediated depletion of NAD+ that could be lethal under certain conditions; prevention of this by SIRT1 inhibition could preserve [NAD+], thus ameliorating cell death [77].

The literature concerning the activation of SIRT1 by RES is similarly controversial. RES was first identified as an activator of SIRT1 in a fluorescent deacetylation assay using a synthetic peptide conjugated to a fluorescent moiety. RES reduced the K_m_ value for the acetylated peptide by 35-fold, significantly altering SIRT1’s substrate binding efficiency [71]. However, subsequent studies revealed that the RES dependent activation of SIRT1 was an artifact of the assay conditions used. Stimulation of SIRT1’s deacetlyation activity with RES was measured in the presence or absence of a nonphysiological Fluor de Lys fluorophore. Three research groups found that RES increases the deacetylase activity of SIRT1 only in the presence of the Fluor de Lys fluorophore [78,79,80]. Using alternative assays that did not involve the Fluor de Lys fluorophore, Borra *et al*., Kaeberelein *et al*., and Beher *et al*. were unable to provide evidence that RES is capable of activating SIRT1 [78,79,80]. Taken together, we believe that the data presented to date are not strongly supportive of a central role for SIRT1 in mediating the neuroprotective activity of RES.

## 5. Increasing RES Bioavailability

While RES possesses a variety of beneficial biological activities, its pharmacological use is greatly limited by difficulties achieving therapeutic concentrations *in vivo*. Following dietary intake RES is rapidly metabolized and undergoes substantial chemical modification in the small intestine [81] or liver [82] and the majority of RES in human plasma following ingestion of the pure compound exists in its 3-O-glucuronide form [83]. On account of its rapid metabolism, supraphysiological doses of RES are required to elevate blood concentrations even slightly [23]. Studies in which rodents have been fed milligram quantities of the isolated compound have measured plasma levels of RES within the nanomolar to low micromolar range 30 minutes to 18 hours following intake [82,84,85]. The experimental concentrations mentioned above greatly surpass those achieved by red wine consumption, the most common dietary source of RES which are typically in the low micromolar range [83]. It may be the case that RES is capable of imparting neuroprotective effects at very low concentrations. Alternatively, tissue specific uptake of RES may be critical to its actions *in vivo*, and it’s bioavailability in tissue may not be predicted by measurements of plasma RES levels alone. Few studies have examined the biological activities of RES derivatives, and it will be interesting to know if these compounds are capable of eliciting similar protective effects.

Recently, Juan *et al*. reported an HPLC method capable of detecting RES in animal tissues at concentrations as low as 5.5 nM. This HPLC method was used to determine RES concentrations in tissues of rats administered intravenous RES at 15 mg/kg for 90 minutes. The accumulation of RES was lowest in brain, reaching only 0.17 +/- 0.04 nM/g tissue [86]. Interestingly, free RES comprised the greatest proportion of detected species in brain tissue, suggesting that the free form of RES is capable of crossing the blood brain barrier. In addition to evidence supporting the presence of RES in brain, El-Mohsen *et al*., found that the rate of RES clearance from brain tissue is substantially lower than in other organs. RES concentrations were measured via HPLC analysis of a methanol extraction from tissue two hours and 18 hours after RES administration. At 18 hours, the RES concentration decreased to only 43% of their initial measurement made two hours following RES administration. In comparison, RES concentration at 18 h fell to only 10% of the initial measurement in liver [82]. It is important to note that RES distribution in tissues and plasma is typically measured following a single dose of the compound. In contrast, many of the studies reporting RES’s neuroprotective effects entail chronic, daily supplementation. Currently, little is current known regarding the potential for RES accumulation in tissues in studies involving long term, daily RES intake and elucidating the effect of chronic RES administration on tissue levels, particularly in brain, is an important next step in understanding the *in vivo* effects of this compound.

An equally important step in developing the therapeutic potential of RES is investigating how the delivery matrix can influence biological activities. Our previous work investigated the ability of three delivery methods to modulate tissue levels of antioxidant enzyme activities in mice: 1) RES incorporated into standard mouse chow; 2) RES incorporated into a high fat mouse chow and 3) RES in DMSO via a subcutaneous osmotic minipump. Delivery in a high fat diet elicited greater changes in cellular antioxidant activities in brain than the other two methods [59]. While the exact mechanisms that underlie the delivery-specific effects are unclear, it has been proposed that the inclusion of RES in a high fat diet protects it from degradation [87]. Alternative delivery systems that protect RES in the digestive tract without the negative consequences of a high fat diet may offer a new way to realize RES’s positive effects on human health. 

Carrier-mediated delivery of RES, including the use of nanoparticles is one potential means by which the challenges facing RES’s bioavailability may be overcome. Two challenges facing the pharmaceutical development of RES are its instability and low solubility in water. Carriers that can modify these characteristics by encapsulating RES in a hydrophobic and stabilizing environment may offer a solution to RES’s low bioavailability. The use of liposomal formulas to deliver other antioxidants molecules such as glutathione, vitamin E and *N*-acetylcysteine has been previously reported with protective effects against disorders associated with oxidative stress [88]. Kristl *et al*., reported the development of a liposome carrier for RES that showed a protective effect against UV irradiation of human derived renal endothelial cells, using the reduction of MTT as an end point [89] While the results of the MTT assay are not necessarily representative of cell viability the RES-liposomes appear to elicit a variety of changes that are not observed in the control groups [89]. The further development of methods to increase tissue concentrations of RES, particularly in brain, will be an important step in realizing the neuroprotective potential of this compound.

## 6. Conclusions

There is now compelling evidence that dietary administration of RES can confer neuroprotection in rodents, raising the exciting possibility that it will prove to be similarly active in humans. The molecular mechanisms underlying RES’s neuroprotective activities remain to be conclusively determined. Experiments with cultured cell lines suggest that the ability to selectively upregulate mitochondrial antioxidant gene expression, particularly MnSOD, may be important. This effect is achievable *in vivo* in mice, providing sufficiently high levels of RES are delivered in an appropriate dietary matrix. An important cellular target of RES may be ERβ, due to RES’s structural similarity to E2. An agonistic interaction with ERβ would explain how circulating RES concentrations that may be quite low (high nanomolar to low micromolar range) are capable of eliciting a significant antioxidant effect in brain tissue. Presumably, strategies capable of increasing RES bioavailability will enhance this desirable effect. Continued study using *in vitro* and *in vivo* experimental models will be required to achieve this goal.
